# Zirconia in fixed prosthesis. A literature review

**DOI:** 10.4317/jced.51304

**Published:** 2014-02-01

**Authors:** Rubén Agustín-Panadero, Juan L. Román-Rodríguez, Alberto Ferreiroa, María F. Solá-Ruíz, Antonio Fons-Font

**Affiliations:** 1 Associate Lecturer. Department of Dental Medicine, Faculty of Medicine and Dentistry, University of Valencia, Spain; 2 Collaborating Lecturer. Department of Buccofacial Prosthesis, Faculty of Odontology, Complutense University of Madrid; 3 Assistant Lecturer. Department of Dental Medicine, Faculty of Medicine and Dentistry, University of Valencia, Spain; 4 Senior Lecturer. Department of Dental Medicine, Faculty of Medicine and Dentistry, University of Valencia, Spain

## Abstract

Statement of problem: Evidence is limited on the efficacy of zirconia-based fixed dental prostheses. Objective: To carry out a literature review of the behavior of zirconium oxide dental restorations. Material and Methods: This literature review searched the Pubmed, Scopus, Medline and Cochrane Library databases using key search words “zirconium oxide,” “zirconia,” “non-metal restorations,” “ceramic oxides,” “veneering ceramic,” “zirconia-based fixed dental prostheses”. Both in vivo and in vitro studies into zirconia-based prosthodontic restoration behavior were included. Results: Clinical studies have revealed a high rate of fracture for porcelain-veneered zirconia-based restorations that varies between 6% and 15% over a 3- to 5-year period, while for ceramo-metallic restorations the fracture rate ranges between 4 and 10% over ten years. These results provoke uncertainty as to the long-term prognosis for this material in the oral medium. The cause of veneering porcelain fractures is unknown but hypothetically they could be associated with bond failure between the veneer material and the zirconia sub-structure.

** Key words:**Veneering ceramic, zirconia-based ceramic restoration, crown, zirconia, tooth-supported fixed prosthesis.

## Introduction

Prosthodontic treatments have traditionally sought to restore lost function (chewing, speech, swallowing), while providing esthetics that fulfill contemporary criteria for attractiveness. The demand for optimum esthetics is conditioned both by social pressure and the interests of the dental profession. Only a few decades ago, some dental restoration types, such as fenestrated crowns or partial coverage crowns, were described as esthetic and in certain ambits demand for these restorations remains high. However, at the present time the term ‘esthetic restoration’ refers to ceramic restorations and in particular to porcelain restorations without any metal. Towards the end of the last century, a climate of non-acceptance of metal alloys in the mouth emerged among some dentists and in the dental product industry and, given the increasing demand for esthetic treatments, these factors have driven the development of new all-ceramic prosthetic rehabilitations. For this reason, recent research (1-8) has focused on ceramics, seeking restorations that provide optimum esthetics while replacing ceramo-metallic restorations with all-ceramic restorations of similar mechanical strength.

## Material and Methods

An exhaustive search of literature published 1995 to 2013 was made in on-line databases (Medline, Pubmed, Scopus and the Cochrane Library) using the following key search terms: *“zirconium oxide”, “zirconia”, “non-metal restorations”, “ceramic oxides”, “veneering ceramic”, “zirconia-based fixed dental prostheses”*. All the articles identified had been published in international scientific journals (Journal Citation Reports). Both in vitro and in vivo studies of the performance of zirconia-based fixed dental prostheses were included. The articles were then evaluated for inclusion in the review by five researchers working independently, applying the following inclusion criteria: randomized and non-randomized controlled clinical trials; in vitro trials of mechanical behavior; systematic reviews; meta-analyses; cohort and case-control studies. Isolated clinical case reports, articles expressing opinion, articles lacking scientific evidence or motivated by commercial interests or sponsorship were discarded. A total of 225 articles were identified in the initial search, of which 177 were discarded for failing to meet the inclusion criteria described above. Information contained in the remaining articles was collated for comparison and analysis.

## Literature Review Results

The endeavor to replace the metal in ceramo-metallic restorations with high-resistance ceramics began towards the end of the twentieth century and has not yet reached a conclusion. At present, zirconium oxide is the main focus of research and clinical trials. The principal characteristics favoring its use as a biomaterial are chemical and dimensional stability, mechanical resistance, hardness, and an elastic modulus of the same order as stainless steel (1).

Zirconium oxide has been in use since 1960. From the start, its promising *in vitro* properties attracted the attention of dental (and orthopedic) researchers and in the last decade it has acquired increasing prominence. The properties that favor its use in dentistry are biocompatibility, low thermal conductivity, resistance to corrosion and high tenacity, due to its totally crystalline microstructure. However, being opaque, it has to be covered with a more translucent feldspathic ceramic to improve esthetics.

When the function of restorations, both all-ceramic and metal-ceramic, is evaluated over time, there are two concepts that are often regarded as synonymous: success and survival. The survival of a restoration means that it fulfills its function in the mouth even though it may have suffered some additional affectation. Success can be defined as a restoration that survives intact maintaining surface qualities, anatomical shape and function, as well as optimum esthetics (1,2).

In zirconia-based fixed dental prostheses, in spite of the material’s high fracture resistance, the porcelain-veneered can chip during mastication and this is a frequent problem (3,4). This complication generates some uncertainty as to the long-term performance of the material’s use in dental restorations (5).

**Figure 1:**
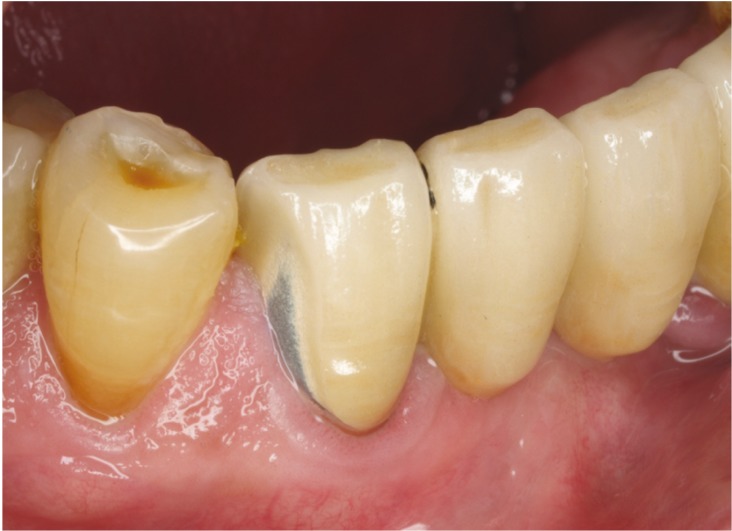
Chipping of ceramic veneer on ceramo-metallic restoration.

Clinical studies have revealed a high rate of fracture for porcelain-veneered zirconia-based restorations that varies between 6% and 15% over a 3- to 5-year period. These are high values compared to the 4% fracture rate shown by conventional metal-ceramic restorations over 10 years (6) (Fig. 1). The cause of these fractures is unknown but might be associated with bond failure between the porcelain-veneered and the zirconia structure (7).

**Figure 2:**
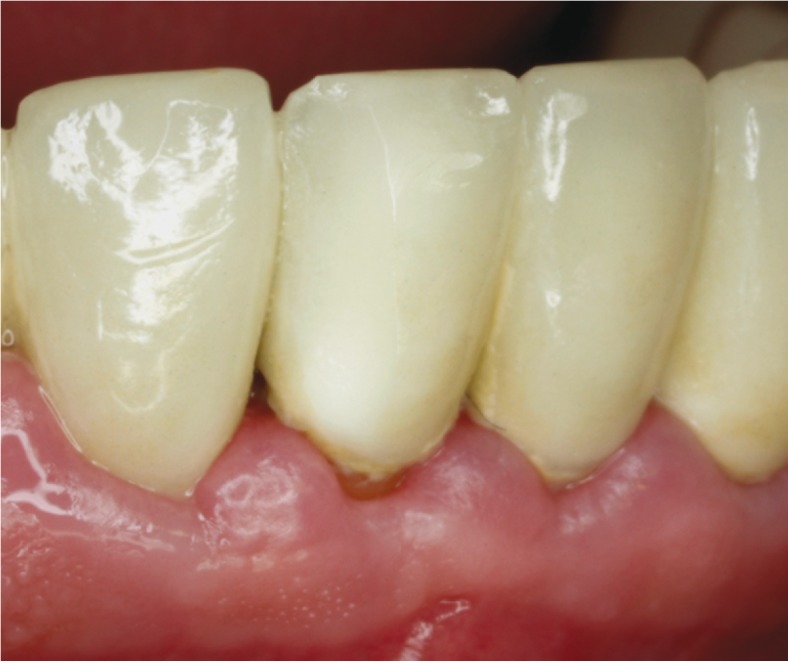
Grade 1 Chipping of a zirconia full-coverage crown (Tooth 41).

According to Heintze and Rousson, the chipping of porcelain-veneered can be classified by severity and the treatment required for repair as follows:

•Grade 1: Small surface chipping. Treatment: polishing the restoration surface (Fig. 2).

**Figure 3:**
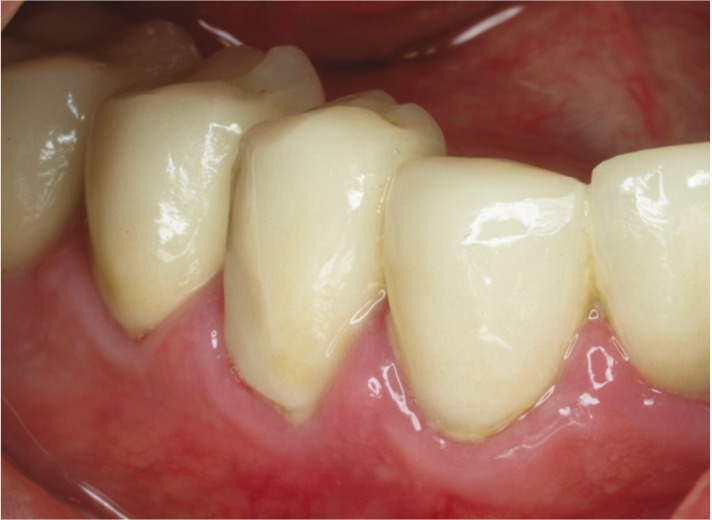
Grade 2 of a zirconia full-coverage crown (Tooth 44).

•Grade 2: Moderate surface chipping. Treatment: use of a resin composite repair system. (Fig. 3)

•Grade 3: Severe veneer ceramic chipping exposing the zirconia core. Treatment: replacement of the damaged prosthesis (8).

Literature reviews such as those made by Raigrodski, Anusavice and Heintze show that the most frequent types of zirconia-based fixed dental prostheses chipping are Grades 1 and 2, which do not involve restoration failure (5,8,9).

Factors that reduce the strength of porcelain-veneered zirconia-based restorations and so increase the risk of chipping are.

•Residual stress caused by differences in the coefficient of thermal expansion (CTE) between the zirconia core and the porcelain-veneered.

•Poor core wettability by the porcelain-veneered, which results in poor engagement between materials and poor micromechanical interlocking.

•Fabrication defects (Griffith defects) (8).

Prosthetic performance is not homogeneous and various factors can influence behavior: the fabrication technique, the extent of the endentulous area between teeth supporting fixed partial prostheses/bridges, or the procedure employed for obtaining the core material. In this way, higher numbers of mechanical failures occur for:

•Traditional manual stratification ceramics than heat-pressed ceramics (8).

•Fixed partial prostheses (bridges) than individual crowns.

•Zirconia restorations fabricated by hard milling of sintered zirconia than by soft milling of pre-sintered zirconia (11).

-Clinical behavior of zirconia-based fixed dental prostheses.

Veneer chipping generally occurs as an esthetic defect of little importance and is easily corrected by polishing or intraoral repair; it often goes unnoticed by the patient (8). For this reason, the survival rates of zirconia-based fixed dental prostheses and ceramo-metallic restorations are estimated to be equivalent (97-99% over five years) (5).

The highest numbers of complications arising from the use of zirconium oxide in prosthodontic treatments occurs with fixed partial prostheses or bridges. The present literature review identified numerous clinical studies in which cohesive fracture of the veneer material is the main and most frequent fault. Nevertheless, there is some controversy as to the frequency of this mechanical failure due to variations in the variables analyzed in different studies ( Table 1):

**Table 1:**
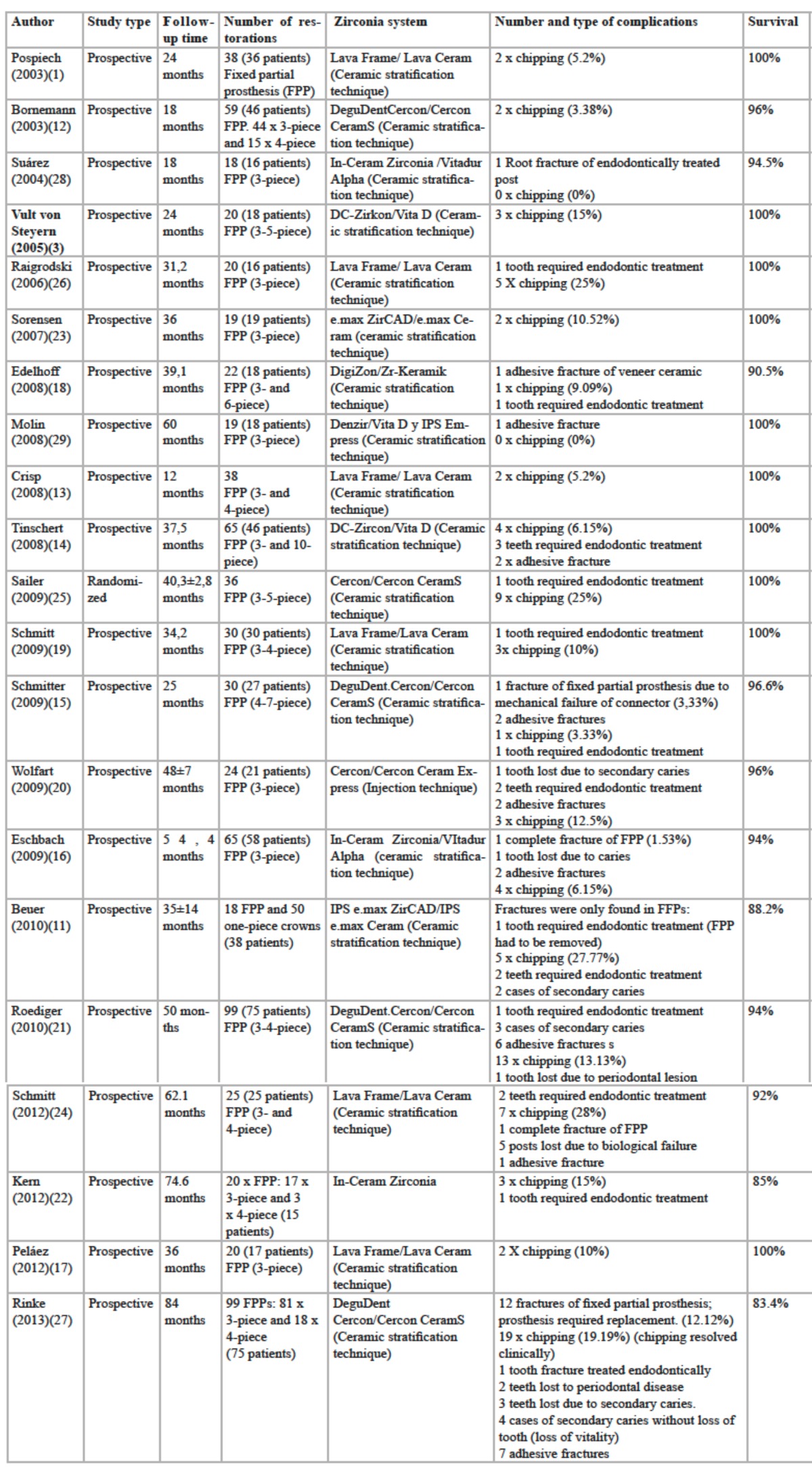
Clinical studies with tooth-supported fixed partial prostheses with zirconia core.

•Pospiech (24-month follow-up), Beuer (40 months), Bornemann (18 months), Crisp (12 months), Tinschert (37.5 months), Schmitter (25 months), and Eschbach (54.4 months) found chipping percentages ranging between 3% and 6% (10,11,12-16).

•Vult von Steyern (24-month follow-up), Peláez (36 months), Edelhoff (39.1 months), Schmitt (34.2 months), Wolfart (48±7 months), Roediger (50 months), Kern (74.6 months), and Sorensen (36 months) carried out in vivo studies of posterior fixed partial prostheses finding an incidence of chipping ranging between 9% and 15% (3,17-23).

•Lastly, diverse *in vivo* studies by Raigrodski (31.2-month follow-up), Sailer (40.3±2.8 months), Beuer (35±14 months), Schmitt (62,1 months) and Rinke (84 months) claim that the incidence of chipping of the veneer ma-terial on posterior fixed partial prostheses ranges between 19% and 28% (24,11,25-27).

Notably, some authors – Molin (60-month follow-up) and Suárez (18 months) – did not detect any mechanical complications at all among the restorations studied (28,29).

**Figure 4:**
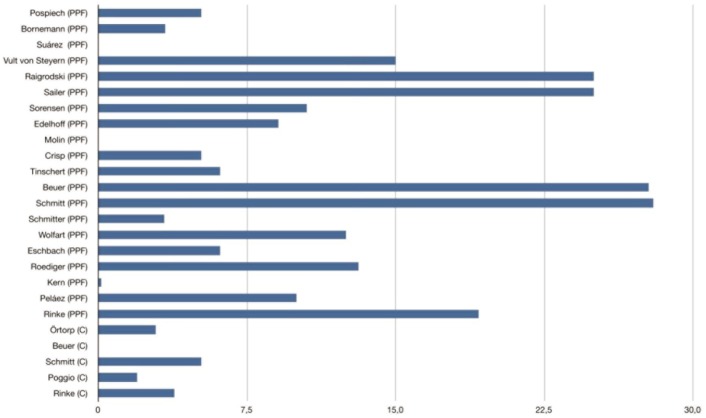
Percentage of chipping/delamination of ceramic veneers in fixed partial prostheses with zirconia cores. Fixed partial prosthesis (FPP); Full-coverage crown (C).

The few *in vivo* clinical studies available in the literature of crown with zirconia substructures – Beuer (35±14 months), Örtorp (60-month follow-up), Poggio (20.9 months) and Rinke (36.5±6 months) – reveal different behavior from fixed partial prostheses, with an incidence of chipping ranging from 0% to 4% (11,30-33) ( Table 2) (Fig. 4).

**Table 2:**
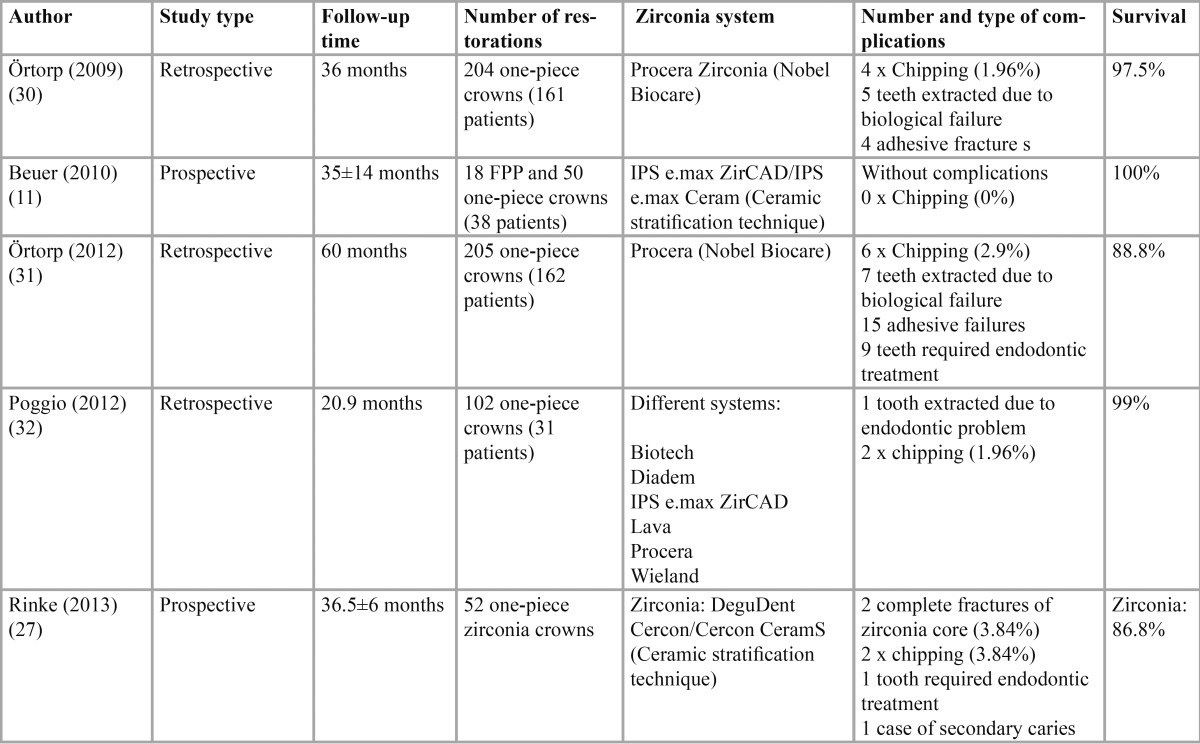
Clinical studies with tooth-supported one-piece full-coverage restorations and inlays with zirconia core.

-In vitro behavior of fixed prostheses with zirconia substructure.

Regarding the mechanical behavior of fixed prosthetic restorations, the most important requirement is that they must withstand mastication forces without fracturing. The first molar is subjected to forces of approximately 300-800 N, while the anterior zone is subjected to mastication forces of 60-200N. In some parafunctional cases occlusal forces can reach 1000 N (34).

According to the literature, compression and flexion trials with vertical and perpendicular vectors would appear to be adequate for testing the fracture resistance of crowns or bridges. In static compression load trials of all-ceramic restorations, the forces applied in different the studies reviewed are as follows (in increasing order): IPS Empress, 130-180 Mpa; In Ceram espinel, 250-350 Mpa; IPS Empress 2, 200-400 Mpa; In-Ceram Alumina, 400-600 Mpa; In Ceram Zirconia, 570-630 Mpa; Procera AllCeram (alumina), 600 Mpa; zirconia-based fixed dental prostheses (Lava, Procera Zirconia, Everest or IPS e.max ZirCAD), 900-1200 MPa (34-47).

Agustín et al. analyzed the behavior of three zirconia-based restoration types subjected to compression loading (Lava, IPS emax ZirCAD, IPS emax ZirPress); the crowns surpassed the forces deemed necessary for clinical survival (1325.7-2310.5 N) (34).

Potiket carried out compression load testing of 40 full coverage crowns, dividing these into groups according to the core material: ceramo-metallic restorations; zirconia (Procera AllZirkon); aluminum oxide (Procera AllCeram). These were subjected to static compression loading; no statistically significant differences in fracture resistance were found between the restoration types (2).

Tsalouchou made a study of 50 zirconia crowns, comparing fracture resistance of two types of veneer ceramic: injected ceramic (IPS e.max ZirPress) and stratified ceramic (IPS e.max Ceram) over zirconia cores. Mean resis-tance for the groups was: ZirCAD+ZirPress (2135.6 ±330.1 N) and ZirCAD+IPS e.max Ceram (2189.9±317.6 N), without statistically significant difference (35).

Agustín et al. made an in vitro study of the mechanical resistance of veneer ceramic on 120 crowns with either metal or zirconia cores, subjecting them to static compression loading: IPS e.max ZirCAD/IPS e.max Ceram (1773.9 N); IPS e.max ZirCAD/IPS e.max Zirpress (1818 N); Lava Frame Zirconia/Lava Ceram (2211 N); Cromo-Niquel/IPS d.Sign (2310.5 N); Cromo-Niquel/IPS InLine (1933.2 N); Cromo-Niquel/IPS InLinePoM (1325.7 N). Zirconia-based restorations IPS e.max ZirCAD, with either injected ceramic veneers (IPS e.max Zirpress) or stratified veneers (IPS e.max Ceram) were statistically less resistant than d.Sign nickel-chromium/IPS and Lava Frame Zirconia/Lava Ceram crowns. Notably, the group that presented the lowest resistance values was Nickel-chromium/IPS InLinePoM (metal-ceramic with injected ceramic veneer), which was significantly less resistant than the other crowns tested (36).

Studies of zirconia-veneer ceramic bond strength subjected to shear forces (lateral loading of specimen to eva-luate resistance to debonding at the zirconia-porcelain interface) were also reviewed. López-Mollá et al. studied six groups: d.SIGN nickel-chromium (13.45 MPa); IPS e.max Press/IPS e.max Ceram (24.20 MPa); IPS e.max ZirCAD/IPS e.max ZirPress (12.70 MPa); IPS e.max ZirCAD/IPS e.max Ceram (7.86 MPa); Lava Frame/Lava Ceram (10.20 MPa); Lava Frame/IPS e.max Ceram (4.62 MPa). The assay applied a lateral static load to the core-ceramic interface with specimens mounted in test cylinders (dimensions: 15mm long x 8mm diameter). It was found that pressure injection molded veneer ceramics (IPS e.max ZirCAD/ IPS e.max ZirPress) bonded more successfully to the zirconia core than veneers applied using stratification techniques or sintering in layers (37).

Choi compared the fracture resistance of porcelain veneers (45 samples) of two restoration types (metal-ceramic and zirconia [Cercon]). The metal-ceramic restorations were significantly more resistant (35.87±4.23 MPa) than the zirconia restorations (25.43±3.12 MPa) (38).

Blatz studied the mechanical behavior of the veneer-core bond of 120 samples (dimensions: 10mm x 10mm x 2mm). Ninety specimens were fabricated with a Lava Zirconia core and divided into three groups according to the veneer (Cerabien ZR, GC Initial and Lava Ceram); a further 30 specimens had a metal core (Control Group). Shear forces were applied to the veneer-core interfaces; resistance was significantly greater for the zirconia groups than the control group (with metal core). For the zirconia samples, all fractures took the form of chipping, pointing to an optimum bond between the zirconia core and the ceramic veneer (39).

Analyzing studies of the fracture resistance of all-ceramic partial fixed prostheses, Rosentritt et al. published mean fracture values of 1500 N for bridges in posterior sectors subjected to cyclic loading (47). Another study (41) obtained fracture resistance values for Lava three-piece bridges of 1816 N, although these were not subjec-ted to cyclic loading. Stiesch-Scholz et al. found significant differences between Lava (1250 N) and Empress 2 (400 N) and showed how cyclic loading produced a reduction in fracture resistance of four-piece bridges for both materials (42). Ludwig et al. compared Empress 2 bridges, which suffered complete fracture when subjected to 729 ± 59 N, with Lava bridges, which suffered ceramic veneer fracture at 848 ± 68 N, obtaining a significant difference (43). Silva et al. tested Lava crowns, obtaining values of 1134 ± 182 N, this study regarded fracture of the ceramic veneer as prosthetic failure, even though the core remains intact (44).

In most of these studies of the mechanical behavior of fixed partial prostheses, fractures occurred that were oblique, from gingival to occlusal, from the connector center to the center of the pontic. For this reason, most authors (40-44) recommend that pontics should be fabricated with an area of 6-9 mm2.

According to Konstantinos and Agustín (34,46), restoration fracture types can be classified as:

•Cohesive (chipping): when the fracture occurs in the porcelain-veneered without affecting the ceramic-core interface.

•Adhesive: when the fracture occurs at the ceramic-core bond.

When samples fracture, most in vitro studies note that the type of fracture suffered by zirconia restorations follows a cohesive pattern in the occlusal zone adjacent to the point of contact with the antagonist (36,45).

In vitro studies of full-coverage restorations have observed a higher incidence of cohesive fracture for zirconia restorations compared to ceramo-metallic restorations (that show predominantly adhesive fractures) (34). The higher incidence of chipping is explained in a study by Martin Rosentritt (2009) that assayed zirconia restoration fracture resistance, finding that all samples suffered cohesive fractures due to inadequate performance of the veneer material. 

Agustín (2012), in a study of ceramic veneer behavior, on zirconia and metal cores, using scanning electron microscopy (SEM) observed that the most frequent fracture type for zirconia-core restorations was cohesive (71.66%), compared to metal core restorations which all showed adhesive fractures (34).

Tsalouchou assayed resistance to static loading of 50 zirconia crowns, making SEM analysis of the transversal plane, also showing that the most frequent fracture type was cohesive (35).

In the same way, Saito made a study of fracture resistance of porcelain-veneered of 72 samples with zirconia cores, finding that the most frequent fracture type was cohesive (88.8%) (48).

To date, no scientific evidence for a chemical union between zirconia and ceramic veneers has been found. The two materials appear to bond by means of mechanical engagement and the formation of compressive strength resulting from thermal contraction during cooling after sintering (34).
